# Design of a prospective follow-up study on early parenthood and smoking behaviour during pregnancy in Finnish primary healthcare

**DOI:** 10.1177/14034948211022433

**Published:** 2021-06-19

**Authors:** Mikael O. Ekblad, Hanna P. Wallin, Marjukka Pajulo, Päivi E. Korhonen

**Affiliations:** 1 Department of General Practice, University of Turku and Turku University Hospital, Finland; 2 Central Satakunta Federation of Municipalities, Finland; 3 Department of Child Psychiatry, University of Turku, Finland

**Keywords:** Primary care, maternity clinic, prenatal exposure, smoking, e-cigarettes, pregnancy, attachment, mentalization, depression, anxiety

## Abstract

*Aims:* The primary aim of the study is to explore different factors affecting parents’ smoking behaviour, and especially how smoking may be connected with individual differences in the psychological process of becoming a parent. In the current paper, we present the study design together with basic information on the study population. *Methods:* The Central Satakunta Maternity and Child Health Clinic (KESALATU) Study is an ongoing prospective follow-up study in primary healthcare of the Satakunta region of southwest Finland. Families were recruited during their first maternity clinic visit between 1 September 2016 and 31 December 2019, and participation will continue until the child is 1.5 years of age. The study combines different sources and types of data: e.g. routine data obtained from primary healthcare clinic records, specific parental self-report data and data from a new exhaled carbon monoxide meter indicating maternal smoking. The data are collected using frequently repeated assessments both during pregnancy and postnatally. The methods cover the following areas of interest: family background factors (including smoking and alcohol use), self-reported parental–foetal/infant attachment and mentalization, self-reported stress, depression and quality of life. *Results:* 589 pregnant women and their partners were asked to participate in the study during the collection time period. The final study population consisted of 248 (42.1%) pregnant women and 160 (27.1%) partners. ***Conclusions*: The new methods and study design have the potential to increase our understanding about the link between early parenting psychology, prenatal psychosocial risk factors and parental health behaviour.**

## Introduction

Maternal smoking during pregnancy is the most common harmful exposure during pregnancy compromising foetal and child health. Smoking during pregnancy has been linked with several well-known adverse effects on pregnancy and foetal outcomes including preterm birth, low birth weight and even neonatal morbidity [1,2]. Maternal smoking disturbs significantly the development of many important organs, especially the central nervous system and lungs of the foetus leading to potential long-term adverse effects, which may result in later psychological and psychological problems [2–4]. Maternal second-hand smoke exposure is also known to have adverse effects on child health [5].

Still one in nine of pregnant women continue to smoke in Finland [6]. It is well established that smoking during pregnancy is more common in women who are young, have low educational level, whose partner is a smoker and who have psychiatric problems [4,7]. Economic stress and socioeconomic status across the mother’s life course are shown to predict maternal smoking [8]. A study by Härkönen et al. showed that socio-economic disparities in smoking during pregnancy are primarily explained by differences in the mother’s level of education [9]. The women whose partners smoke continue to smoke during pregnancy four times more often compared to women whose partners do not smoke [10].

Finland is one of the first countries to set an ambitious goal to end the use of tobacco and other nicotine products, meaning less than 5% of adults consuming tobacco or nicotine products, by the year 2030 [11]. The prevalence of smoking among adults has been decreasing during the last decades and currently 15% of men and 13% of women smoke daily in Finland [12]. Despite the general positive changes in the prevalence of smoking, it is worthy of notice that a similar decrease has not yet occurred among Finnish pregnant women, and especially not during the early pregnancy phase [6–7].

Parental–foetal/infant attachment and parent mentalization might offer new intervention routes for smoking cessation among the parents-to-be. Prenatal attachment refers to the emotional attachment of a parent to the foetus, which is manifested both in parental behaviour and in the content and amount of mental focusing on the foetus [13]. The relationship between the parent and child starts to develop already when a desire for a child exists [14]. The individual process of becoming a parent for the first time is one of the most intense transitional stages in human life. This requires mental preparation and adjustment, and often brings up ambivalent and vulnerable thoughts [14]. The mental representations, the internalized memories of parental practices and experiences from one’s own childhood, become stronger when the mother can feel the movement of the foetus and the baby becomes more real; this starts to strongly shape the quality of the parent–child relationship, that is parent–foetal attachment [15–17].

Mentalization refers to the parent’s capability to think of the child as a separate, individual being from early on [18]. A parent who is capable of mentalizing the baby/child is able to accept the necessary changes in daily lifestyle and rhythm that pregnancy and parenthood bring, as well as being able to imagine both the challenging and the most rewarding postnatal situations with the child [19–20]. A strong mentalization capacity contributes to a stronger parent–foetus/infant attachment and strengthens early interaction with the child [21].

The Finnish maternity and child health clinics offer a good opportunity for supporting smoking cessation and the development of parental attachment. The clinics are run by the municipalities in Finland, and the Ministry of Social Affairs and Health regulate the scheme [22]. The maternity and child health clinic visits are free-of-charge for all residents in Finland, thus, in practice, all pregnant women attend the maternity clinics. On average, expectant women have 11 nurse and/or physician appointments during pregnancy.

There is an urgent need to try to discover how to better enhance smoking cessation among both pregnant women and their partners. Those women who continue to smoke during pregnancy most likely suffer from stronger nicotine dependency and have also other challenges in their life management. This study has the potential to find new intervention routes and to gain knowledge about for whom to provide more comprehensive family support. Conducting research in maternity and child health clinics in a primary healthcare setting is an important way to improve current practices that will lead to better health care.

### Overview and aims of the current study

The Central Satakunta Maternity and Child Health Clinic (KESALATU) Study is an ongoing prospective follow-up study on the primary healthcare of the Central Satakunta region of southwest Finland. It was designed to gather comprehensive data on pregnant women and their partners, as well as their children up to the age of 1.5 years. The data are collected using different sources of information, for example maternity and child healthcare clinic records, parental self-reports and a new exhaled breath carbon monoxide (CO) meter assessing amount of tobacco smoking from exhaled air. The primary aims of the study are presented in Table I.

**Table I.** Primary aims of the prospective follow-up Central Satakunta Maternity and Child Health Clinic (KESALATU) study.

The first goal of the study is to gather comprehensive data on the use of alcohol, tobacco and other products containing nicotine in families attending maternity clinics in the Central Satakunta region.
The measurement of exhaled breath CO will be used as an objective measurement of smoking, and it will allow us to examine the effects of CO levels on maternal health during pregnancy and pregnancy outcomes.
The second goal is to examine the factors having an effect on parental smoking habits prior, during and after the pregnancy, including, for example, the role of parents’ experienced stress, partner’s smoking habits and development of prenatal attachment.
We will also be able to explore the importance of timing of these factors, and identify factors predisposing parents to start smoking again postpartum.
The third goal is to explore, how e.g. parental perinatal stress, prenatal smoking and intimate partner violence affect early parenthood in terms of prenatal attachment and/or capacity to mentalize the child.
The fourth goal is to explore the potential of the new pregnancy diary to support parent–foetal attachment and parental mentalization, and to decrease experienced stress and smoking behaviour.

## Material and methods

### Study region

The Central Satakunta Health Federation of Municipalities is responsible for organizing the maternity and child health clinics in the study area. It includes four municipalities, participating in the current study (Harjavalta, Nakkila, Kokemäki and Eurajoki), with a total population of around 30,000 inhabitants.

### Recruitment methods

Families expecting a baby were recruited by the primary healthcare nurses during their first maternity clinic visit between 1 September 2016 and 31 December 2019. The only inclusion criterion was that the parent should speak and understand Finnish fluently. In the study area, the proportion of the Finnish-speaking population was 97% in 2018 [23]. The data collection continues until the last participating child is 1.5 years old, that is until spring 2022.

### Ethics

Written consent was obtained from all participants. The study data collection procedures were performed during normal maternity and child health clinic visits by the primary healthcare nurses. The participants did not receive any rewards for participating in the study. The KESALATU Study protocol was approved by The Ethics Review Committee of the Hospital District of southwest Finland. Based on the current ethical approval, it is possible to merge register data to the study data, though the permission for register data use needs to be further applied from the register-keepers.

### Assessment methods

**Figure 1.**
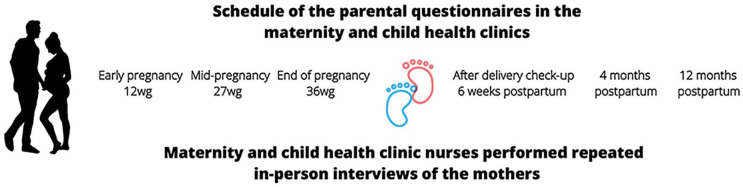
Overview of the Central Satakunta Maternity and Child Health Clinic (KESALATU) Study.

The assessment protocol included repeated (a) interview and measurement data of the mother and the child collected by maternity and child health clinic nurses and (b) parental self-report questionnaires, given to both parents (Figure 1).

#### Interview and measurement data

The maternity clinic nurses performed in-person interviews with the mothers and collected information about the progress of the pregnancy during the routine maternity clinic visits. During the first visit, the nurses collected background information on maternal age, height, weight, previous pregnancies and smoking status within one year before pregnancy. After the delivery, the nurses collected information on the new-born and on pregnancy complications (pre-eclampsia, gestational diabetes, hepatogestosis and pregnancy-related blood pressure). Table II depicts the repeatedly measured and interviewed data during study.

**Table II.** Repeated measurements during the maternity and child health clinic visits in the Central Satakunta Maternity and Child Health Clinic (KESALATU) study.

Maternal variables	Maternity clinic visits (gestation weeks)	12+	18+	22+	27+	30+	36+	After delivery check-up	Child health clinic visits (age, months)	2	4	6	8	10	12	18
	8+								1							
Maternal weight (kg)	x	x	x	x	x	x	x	x			x	x			x	
Fundal height (cm)			x	x	x	x	x									
Blood pressure (mmHg)	x	x	x	x	x	x	x	x								
Protein in urine			x	x	x	x	x									
Smoking (cigarettes/day)	x	x	x	x	x	x	x	x	x	x	x	x	x	x	x	x
CO (ppm)	x	x	x	x	x	x	x	x			x				x	x
Maternal e-cigarette use (yes/no)	x	x	x	x	x	x	x	x	x	x	x	x	x	x	x	x
Child variables																
Weight (g)									x	x	x	x	x	x	x	x
Weight-% (%)									x	x	x	x	x	x	x	x
Height (cm)									x	x	x	x	x	x	x	x
Head circumference (cm)									x	x	x	x	x	x	x	x
Lactation status (breastfeeding/baby formula/both)									x	x	x	x	x	x	x	

#### Parental questionnaires

The questionnaires included (a) detailed questions about socio-demographic factors, (b) use of alcohol, tobacco and other nicotine products, and (c) standardized self-report questionnaires which assess the areas of specific interests: parental–foetal/infant attachment [24–27], mentalization [19,28], depression [29,30], anxiety and stress [31], and quality of life [32,33] during and after pregnancy. Table III depicts the schedule and published reliability data of the parental questionnaires and assessments.

**Table III.** Published reliability data and schedule for the questionnaires in the The Central Satakunta Maternity and Child Health Clinic (KESALATU) Study assessment protocol.

Domain	Assessment method	Published reliability data	Short-term test-retest	Maternity clinic	Mid-pregnancy (27 wg)	End of pregnancy (36 wg)	After delivery check-up (6 wk postpartum)	Child health clinic	6 months postpartum	12 months postpartum	18 months postpartum
		Coefficient alpha (internal consistency)		Early pregnancy (12 wg)				4 months postpartum			
Parental questionnaires											
Questionnaire for background information and current use of tobacco, other nicotine products, and alcohol				M/F	M/F	M/F	M/F	M/F		M/F	
Smoking	Heaviness of Smoking Index (HSI) [34]	0.72		M/F	M/F	M/F	M/F	M/F		M/F	
Alcohol use	Alcohol Use Disorders Identification Test – 3 item (AUDIT-C) [35]	0.78–0.81		F	F	F	F	M/F		F	M
Study related assessments											
Parent–foetal/infant attachment	Maternal/paternal antenatal attachment scale (MAAS/PAAS) [24,25]	0.82		M/F	M/F	M/F					
	Maternal/paternal postnatal attachment scale (MPAS/PPAS) [26,27]	0.78–0.79	0.70				M/F	M/F		M/F	
Mentalization	Prenatal Parental Reflective Functioning Questionnaire (P-PRFQ) [19]	0.57			M/F	M/F					
	Postnatal Parental Reflective Functioning Questionnaire (PRFQ-Fi) [28]	0.78					M/F	M/F		M/F	
Depression	Edinburgh Postnatal Depression Scale (EPDS) [29,30]		0.92	M/F	M/F	M/F	M/F	M/F		M/F	
Anxiety	Perinatal Anxiety Screening Scale (PASS) [31]	0.96	0.74	M/F	M/F	M/F	M/F	M/F		M/F	
Quality of life	EUROHIS quality of life 8 item index (EUROHIS- QOL 8) [32]	0.78		M/F		M/F		M/F		M/F	
Routinely collected questionnaires [52]											
Everyday resources	Everyday resources for families expecting their first child				M/F						
	Everyday resources for families with a new-born baby							M/F			
	Everyday resources for families with a small child										M/F
Violence	Domestic violence enquiry and assessment form				M/F						
	Violence enquiry for couples								M/F		

All parental questionnaires filled by both mothers and fathers. Routinely collected questionnaires filled in by mother, father, or both.

F, father; mo, month; M, mother; wg weeks of gestation; wk, week.

##### Assessment of mentalization

Assessment of mentalization has previously only been possible using semi-structured and demanding interview methods. Recently, new self-report methods have been developed in international co-operation, which enable collecting large data [19,28]. Quality of life was measured with the EUROHIS Quality of Life 8-item index (EUROHIS-QOL 8), which is an abbreviated and translated version of the WHOQOL-BREF [32,33]. The items are composed of overall quality of life, general health, energy, daily life activities, esteem, relationships, finances and home. Translations of the self-report measures: The Parental Reflective Functioning Questionnaire, Edinburgh Postnatal Depression Scale, EUROHIS-QOL 8, Heaviness of Smoking Index, and 3-item Alcohol Use Disorders Identification Test questionnaires have validated versions in Finnish. The Perinatal Anxiety Screen Scale and parental foetal and infant attachment questionnaires underwent an official translation/back-translation process to adapt them to the Finnish language. The back-translated versions were lastly sent to the original developers of the questionnaires, in order to contrast its conceptual content-based equivalence. The study has access also to questionnaires regarding everyday resources of the families and family violence, which are routinely collected during maternity and child health clinic visits.

##### Parental smoking status

Parental smoking status was first asked by the question ‘Have you smoked within one year of pregnancy?’ (no/quitted smoking/still smoking). Current smokers were asked to fill in the Heaviness of Smoking Index [34] and to report the number of cigarettes (a) per day or (b) per week. The use of e-cigarettes was asked about by the question ‘Do you use e-cigarettes daily?’. Use of alcohol during pregnancy was categorized for expectant mothers in the following way: no alcohol use/quitted when planning pregnancy/quitted when pregnancy was known/yes, current use (average doses per week). For fathers we included the 3-item Alcohol Use Disorders Identification Test [35] in all questionnaires.

##### The ‘Baby in Mind’ week-to-week pregnancy diary

This diary has been developed by a multidisciplinary team of clinicians and researchers from Folkhälsan, Turku University Hospital and the University of Turku, in co-operation with the Counselling Development Centre from the Department of Health and Welfare, THL. It focuses on the child’s perspective from an early stage of parenting and aims to increase the motivation to take care of one’s own and the baby’s health and well-being [17]. The ‘Baby in Mind’ pregnancy diary is available to all Finnish families expecting a baby via an electronic link [36]. A short section in the first postpartum questionnaire asks, whether and how the mother/family has used the pregnancy diary.

### Exhaled breath CO

The routine use of exhaled breath CO meters began in the maternity clinics of the study area in March 2016. Thus, we have access to routine exhaled CO measurements, a biochemical marker of smoking, throughout the pregnancy. The exhaled breath CO meter offers an objective and non-invasive measurement of smoking giving instant feedback, which best suits routine maternity clinic work. Using a cut-off point of 4 parts per million (ppm) during pregnancy [37], the sensitivity of CO meters has ranged between 90 and 96%, with specificity of 92%, for those who have been smoking within 24 hours. In this study, we used the exhaled breath CO meter, the Carefusion BabyCO Monitor, which is a commercially available tool. For the study population, the CO measurement was performed three times at each visit (Table IV), and the maximum CO level was recorded. The mother was classified as non-smoker even if the CO levels were above 4 ppm if (a) the mother declared no smoking in the nurse interview despite the elevated CO levels (six women, CO level ranged from 5 to 9 ppm) and (b) self-reported no smoking in the parental assessments.

**Table IV.** Characteristics of the Central Satakunta Maternity and Child Health Clinic (KESALATU) study population at the first trimester of pregnancy in Finnish primary healthcare in years 2016–2019.

	Pregnant women		Partners	
	*n* (%)^a^	Total *n*	*n* (%)^a^	Total *n*
**Age, mean (SD)**	28.9 (4.7)	247	31.4 (5.9)	156
< 25	47 (19.0)		15 (9.6)	
25–34	164 (66.4)		99 (63.5)	
35 or more	36 (14.6)		42 (26.9)	
**Parity**		232		
0	98 (42.2)			
1 or more	134 (57.8)			
**Marital status**		216		
Married/cohabiting	203 (94.0)			
Single	13 (6.0)			
**Education**		218		156
Under 9 years	16 (7.3)		19 (12.2)	
9–12 years	109 (50.0)		106 (67.9)	
Over 12 years	93 (42.7)		31 (19.9)	
**Occupation**		215		157
Full-time job	163 (75.8)		144 (91.7)	
Entrepreneur	21 (9.8)		6 (3.8)	
Part-time job	13 (6.0)		0 (0)	
Unemployed	18 (8.4)		7 (4.5)	
**Yearly income of the family**		194		
under 30,000 euros	30 (15.5)			
30,000–49,999 euros	51 (26.3)			
50,000–74,999 euros	81 (42.7)			
over 75,000 euros	32 (16.5)			
**Smoked within one year of pregnancy**	77 (31.2)	247	56 (35.4)	158
**Smoking during first trimester**	33 (13.4)	246	49 (31.0)	158
Number of cigarettes/day, mean (min, max)	5.7 (1, 15)	28	11.8 (1, 30)	45
Maximum CO level, mean (min, max)	6.0 (0, 23)	29		
HSI among smokers, mean (SD)	0.9 (0.9)	24	1.4 (1.3)	47
**Use of e-cigarettes**	2 (0.8)	245	8 (5.1)	157
**Use of Swedish snuff**	0 (0)	217	14 (8.9)	157
**Alcohol use**		217		
No alcohol use	46 (21.2)			
Quitted when planning pregnancy	51 (23.5)			
Quitted when pregnancy was known	120 (55.3)			
Yes, currently using	0 (0)			
**AUDIT-C, mean (SD)**			4.0 (1.9)	157
**Illicit drug use**	0 (0)	217	0 (0)	158

a if not stated otherwise.

AUDIT-C, 3-item Alcohol Use Disorders Identification Test; HIS, heaviness of smoking index.

### Statistical power and planned analyses of the KESALATU Study

The aim was to collect comprehensive data that covers multiple areas of interest on families during pregnancy and the first 1.5 years of offspring’s life. Information on parental smoking and the use of other nicotine products have been gathered robustly throughout the follow-up, which enables us to assess the timing of smoking and change in smoking habits. Parental health surveys were conducted repeatedly to assess changes in parental well-being. In addition, comprehensive background information on the parents was gathered in the parental questionnaires. Our rich dataset includes categorical as well as continuous variables, mainly derived from questionnaire data, which can be analysed either as a continuous variable or as a categorical variable. There are several research questions (primary aims in Table I), and multiple statistical methods will be used in the analysis of the data, thus a detailed description of each of the analyses is not possible within the scope of this paper. In the following chapters, we present the main plans briefly.

First, we will examine how the background factors of the study population differ according to smoking status, but also according to other factors, for example alcohol use stopped when planning the pregnancy v. after the positive pregnancy test result/planned v. unplanned pregnancy/parental stress/parental mentalization capacity. We will evaluate the statistical differences between binary variables by using the χ^2^ test. For continuous variables, independent sample *t*-test or Mann–Whitney U-test will be used depending on normality assumption.

Second, we will explore, how, for example, parental perinatal stress, smoking habits and intimate partner violence affect development of prenatal attachment and mentalization capacity. A multi-way analysis of co-variance for repeated measures will be performed to analyse the relationship between prenatal attachment and mentalization capacity and independent variables. F values and degrees of freedom, and 95% confidence intervals (CI) for estimated means will be calculated. Normality assumption will be checked with a Shapiro Wilks test.

Third, factors affecting the prevalence and changes in, for example, smoking habits or the use of other nicotine containing products of the parents will be studied. For example, we will examine how the development of prenatal attachment is related to parental smoking cessation before pregnancy or at different stages of pregnancy by using multivariate logistic regression to calculate odds ratios. The relationship between parental mentalization capacity or perceived stress and changes in parental smoking habits/alcohol use can be studied similarly.

All analyses will be adjusted with appropriate confounding factors. The statistical analyses will be performed using commercially available software (SAS, version 9.4; SAS Institute Inc, Cary, NC).

The statistical power of the study was estimated by using the SAS software. Sample size calculations were formed based on the primary question regarding the association of smoking with the development of prenatal attachment and were calculated separately for mothers and fathers. We used in the calculation a *t*-test with a force of 80% and a two-sided significance level of 0.05. The primary question was if there is a difference between development of prenatal attachment for those who have smoked before/during early pregnancy compared to non-smokers. For mothers, the standard deviation per group was estimated to be seven and for fathers to be eight, and the desired difference between groups was estimated to be five points. In this case, there should be 32 mothers per group and 42 fathers. The cohort has enough statistical power that statistically significant risk estimates can be expected for the primary questions.

## Results

A total of 589 pregnant women and their partners were asked to participate in the study, and 277 (47.0%) of the pregnant women and 175 (29.6%) of the partners agreed. Twenty-nine pregnant women had an early drop-out; two due to moving outside the study area and at least 19 due to early miscarriages. Fifteen partners had an early drop-out. The final study population consisted of 248 (42.1%) pregnant women and 160 (27.1%) partners. Thirty pregnant women and two partners did not fill in the first parental questionnaire but continued in the study. Table IV depicts the characteristics of the sample during the first trimester.

The prevalence of smoking among the participating women prior to pregnancy was high (31%), but 57% of them quitted smoking before the current pregnancy (Table IV). Of the partners, 35% smoked prior to the current pregnancy and only 13% succeeded in quitting smoking prior to the pregnancy. Two (1%) pregnant woman and eight (5%) partners reported using e-cigarettes during the first trimester of pregnancy. None of the pregnant women used alcohol after the pregnancy was confirmed. However, 56% had used alcohol until the pregnancy was known.

## Discussion

Smoking during pregnancy is known to cause a significant risk to the course of pregnancy, maternal health and the development of the foetus and child. It is also a risk factor that could be eliminated with more efficient prevention work. Information and awareness of this fact has long been widely available, but nonetheless, smoking during pregnancy is still too often a problem. More effective tools and intervention pathways are needed to reduce parental smoking in pregnant families and families with young children. Further, there is still sparse knowledge on both the individual differences in the psychological process of becoming a parent and the factors influencing the differences, as well as the factors influencing smoking behaviour during pregnancy, and its individual variation. This research design allows us to gain a better understanding of both these factors and their interrelationship.

The KESALATU Study is a prospective follow-up study carried out in maternity and child health clinics in primary healthcare, focusing on early parenthood and smoking behaviour. The study gathers unique research data on information: routinely collected parent and child data from the maternity and child health clinics, and study-specific data collected with repeated self-report measures and a new test battery. It covers multiple areas of interest and includes the use of some entirely new methods and tools. Together they have the potential to provide missing information on the links between early parenting psychology, risk factors, and health behaviours.

Prenatal attachment has been found to be positively associated with postnatal interaction and involvement between mother and infant [21,38], as well as child’s later development [39]. There is significant individual variation in the timing and intensity of the development of prenatal attachment and mentalization. The reasons behind these individual variations are still largely unknown. It is necessary to rigorously examine the possible obstacles, such as specific psychosocial risk factors, including mental health problems or substance addictions, that make such a development of prenatal attachment and mentalization difficult or even impossible [40–42]. Early parenthood can, however, be significantly strengthened by well-targeted interventions even in the most severe risk groups [43]. One such attempt is the new pregnancy diary, which has been designed to increase the parent’s curiosity towards the baby and his/her development and help the parent to think of daily situations from the baby’s perspective from very early on [17].

In previous studies, the information on smoking during pregnancy has usually been based on maternal self-report, although it is known to underestimate the true prevalence of smoking [44]. The golden standard to verify smoking status would be to use, for example, saliva or plasma cotinine [45]. In this study, maternal smoking status is verified by using the measurement of exhaled breath CO. The mean CO levels have been shown to be 5 ppm in passive and 17 ppm in active smokers [46]. It has even been suggested that exhaled CO measurement is better at measuring the consumption and changes in consumption in smoking compared to cotinine measurement [47]. This might be due to the highly variable nicotine metabolism with a half-life of 16–30 hours [48]. A recent study by Reynolds et al. [49], showed that a CO level of 3 ppm or more during the first antenatal visit associated with lower birth weight and more adverse pregnancy events. In this study, parental smoking has been assessed with repeated self-report questionnaires, with interviews conducted by nurses, and with objective measurement of the exhaled breath CO. This makes it possible to comprehensively observe and consider the effect of both the duration and amount of smoking.

Previously, it has been suggested that very few Finnish women stop smoking before pregnancy [7] because the prevalence of general smoking among women and smoking during first trimester of pregnancy have been at a similar level (Figure 1). However, our results showed that the prevalence of smoking prior to pregnancy was surprisingly high as one in three pregnant women smoked but over half of those women quitted smoking prior to the current pregnancy. The use of e-cigarettes during the first trimester of pregnancy is still uncommon among pregnant women in our population, but the prevalence is likely to increase in the future as the use of e-cigarettes is increasing among young people [50]. Reports from the USA show that already 5% of women have used e-cigarettes during pregnancy [51].

A major strength of this study is that it is the first study to collect comprehensive information on early parenting psychology, prenatal psychosocial risk factors and parental health behaviour, including smoking behaviour, from standard customers of maternity and child health clinics in a primary healthcare region. The limitations of the study include rather low participation rate (42% for pregnant women and 27% for partners), which may affect the ability of the study to draw definitive conclusions. No information was collected on non-participating families. We speculate, that the longitudinal design of the study and repeated assessment of smoking by exhaled CO might have been the main reasons for declining to participate in the study. Thus, non-participating families might be in more vulnerable social situation compared to the participating families. Though our study includes comprehensive information on parent’s current education, occupation, family income, it lacks information on parents’ economic stress and socioeconomic status in their childhood, which might be a precursor of smoking later on [8]. The prevalence of maternal smoking during pregnancy in this study population (13.4%) was at the same level as the smoking during early pregnancy (12–14%) in Finland (Figure 1) [6]. Thus, it seems that smoking families have participated satisfactorily in the study. In addition, the characteristics of our study population represent the standard Finnish population attending maternity clinics in terms of maternal age, parity, marital status and smoking [6], which reduces the risk of selection bias in this study.

Conducting research in primary healthcare is important in order to transmit scientific knowledge and to support the development of methods that will deliver better healthcare. Research work may stimulate the functioning of community health centres, make the personnel more committed, provide training and promote the adoption of evidence-based practices. Support from the administration of the healthcare centres and collaborative networks with specialized care and the university are important prerequisites for successful research in a primary care setting.
